# Prognostic Impact of New‐Onset Atrial Fibrillation After Cardiac Arrest: Observations From a Federated Research Network

**DOI:** 10.1111/jce.70288

**Published:** 2026-02-10

**Authors:** Enrico Tartaglia, Tommaso Bucci, Amir Askarinejad, Michele Rossi, Muath Alobaida, Steven Ho Man Lam, Mert Kaskal, Andrea Galeazzo Rigutini, Shir Lynn Lim, Giuseppe Boriani, Gregory Y. H. Lip

**Affiliations:** ^1^ Liverpool Centre for Cardiovascular Science at University of Liverpool, Liverpool John Moores University and Liverpool and Heart and Chest Hospital Liverpool UK; ^2^ Cardiology Division, Department of Biomedical, Metabolic and Neural Sciences Italy University of Modena and Reggio Emilia, Policlinico di Modena Modena Italy; ^3^ Clinical and Experimental Medicine PhD Program University of Modena and Reggio Emilia Modena Italy; ^4^ Internal, Vascular and Emergency Medicine—Stroke Unit University of Perugia Italy; ^5^ Department of Life, Health & Environmental Sciences University of L'Aquila L'Aquila Italy; ^6^ Internal Medicine and Nephrology Division ASL1 Avezzano‐Sulmona‐L'Aquila, San Salvatore Hospital L'Aquila Italy; ^7^ Department of Basic Science, Prince Sultan Bin Abdulaziz College for Emergency Medical Services King Saud University Riyadh Saudi Arabia; ^8^ Department of Pharmacology School of Medicine, Marmara University Istanbul Turkey; ^9^ Department of Cardiology National University Heart Centre Singapore Singapore; ^10^ Department of Medicine National University of Singapore Singapore; ^11^ Pre‐hospital Emergency and Research Centre Health Services and Systems Research, Duke‐NUS Medical School Singapore; ^12^ Department of Clinical Medicine Aalborg University Aalborg Denmark; ^13^ Department of Cardiology, Lipidology and Internal Medicine Medical University of Bialystok Bialystok Poland

**Keywords:** atrial fibrillation, cardiac arrest, cardiovascular outcomes, myocardial infarction, stroke

## Abstract

**Background:**

Data on adverse events in patients developing atrial fibrillation (AF) after cardiac arrest (CA) are limited.

**Methods:**

Retrospective analysis using the TriNetX network. Adults (≥ 18 years) with CA (ICD‐10‐CM I46) from 2014 to 2024 were divided into two cohorts: those with new‐onset AF (I48) within 2 days after CA and those without. Primary outcome was the 1‐year risk of a composite of all‐cause death, stroke, acute myocardial infarction (AMI), and recurrent CA. Secondary outcomes were the individual components. Cox regression provided hazard ratios (HRs) and 95% confidence intervals (CIs) before and after 1:1 propensity score matching (PSM). Sensitivity analyses stratified follow‐up into early (3–33 days) and late (34–367 days) phases; subgroups included age, sex, hypertension, diabetes, heart failure, and shockable rhythms.

**Results:**

We identified 1221 patients with post–CA AF (mean age 65 ± 14.9 years; 33.8% female) and 149 222 without AF (57.9 ± 17.6 years; 39.3% female). Before PSM, AF patients were older and had greater cardiovascular burden. After PSM post–CA AF was associated with higher risk of the composite outcome (HR 1.25, 95% CI 1.13–1.38), AMI (HR 1.59, 95% CI 1.30–1.95), recurrent CA (HR 1.48, 95% CI 1.30–1.70), and a non‐significant trend toward increased stroke risk, which was greater during the early phase post‐CA. Female patients showed stronger association between post–CA AF and stroke (p for interaction = 0.04).

**Conclusion:**

Post–CA AF is associated with an increased risk of adverse events, including AMI and recurrent CA. Stroke risk was higher during the early post‐resuscitation phase and among female patients.

## Graphical abstract

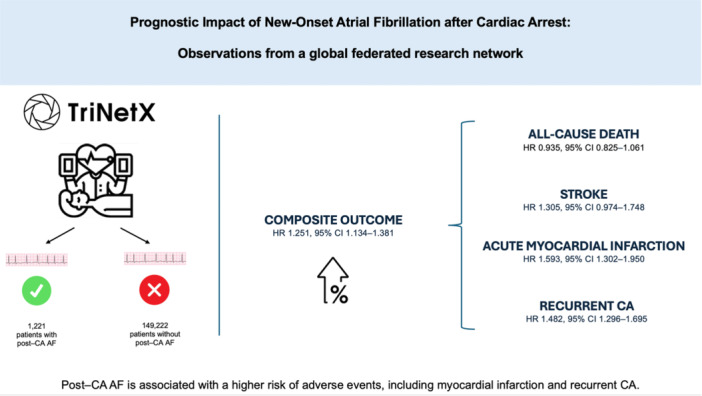

## 1 Introduction

While AF has been extensively studied in chronic cardiovascular settings, its occurrence in acute cardiovascular conditions including scenarios with electrical and hemodynamic instability, such as post–cardiac arrest (CA), has garnered increasing attention in recent years [1].

CA represents one of the most critical events in cardiovascular medicine, often resulting in profound systemic and myocardial stress [2]. The relationship between AF and CA is complex: AF may precede and potentially contribute to CA, particularly in the presence of rapid ventricular response or concomitant structural heart disease [3]. Conversely, it may also emerge as a consequence of the arrest itself, triggered by transient ischemia, autonomic dysregulation, systemic inflammation, or metabolic derangements [1, 4]. Observational studies showed that AF can develop within hours to days following resuscitation from CA, raising important questions regarding its prognostic significance in the post‐resuscitation phase [1].

Although both AF and CA are independently associated with poor outcomes [2, 5], the clinical overlap between the two conditions remains insufficiently characterised. For example, it is unclear whether AF emerging after CA simply interacts with the underlying acute insult—through hemodynamic instability, thromboembolic complications, or arrhythmia‐related recurrence—or whether it independently contributes to the long‐term risk of adverse outcomes [2]. Clarifying this relationship is essential, particularly in a population marked by extreme vulnerability and high early mortality.

The aim of this study was to evaluate the 1‐year risk of adverse events of patients who developed AF following CA, compared to those who experienced CA without subsequent AF, using data from a large global federated research network.

## 2 Methods

### 2.1 Study Design

This retrospective observational study was conducted using the TriNetX Research Network (TriNetX, Cambridge, MA, USA), a global federated health research network that provides access to anonymized electronic medical records from over 130 healthcare organizations, including academic medical centres, community hospitals, and outpatient clinics. The network includes more than 300 million individuals, primarily in the United States (U.S.).

Available data include demographics, diagnoses coded using the International Classification of Diseases, Ninth and Tenth Revisions, Clinical Modification (ICD‐9‐CM and ICD‐10‐CM) codes, and medications coded with Veteran Affairs (VA) codes or RxNorm classification systems, depending on the contributing institution.

Further information about the network is available online (https://trinetx.com/company-overview/).

TriNetX operates in compliance with the Health Insurance Portability and Accountability Act (HIPAA) and US federal law. All patient data are de‐identified in accordance with HIPAA's Privacy Rule, and no patient‐level identifiers are accessible. Access to the data is governed through formal data‐sharing agreements with participating institutions. As a federated network using only de‐identified data, studies conducted within TriNetX do not require approval from institutional review boards or informed consent. Additional methodological details are provided in the Supporting Information S1: Materials.

### 2.2 Cohort Definition

The searches on the TriNetX online research platform were performed on June 24, 2025, using the U.S. Collaborative Network. Based on recorded ICD‐10‐CM, patients aged 18 years or older were included if they had a recorded diagnosis of CA (ICD‐10‐CM: I46) between January 1, 2014, and January 1, 2024.

Two mutually exclusive cohorts were constructed. (1) The first cohort (Post‐CA AF) included patients who developed a new diagnosis of AF (ICD‐10‐CM: I48) within 2 days on or after the first instance of CA. (2) The second cohort (No Post‐CA AF) included patients with CA who did not develop AF within the 2 days following the event. Patients with any documented history of AF prior to the index CA were excluded from both cohorts. The index event was defined as the first recorded diagnosis of CA during the study period. The 2‐day window used to define post–CA AF was selected a priori based on evidence demonstrating that AF occurring within the first 48 h after resuscitated CA carries important prognostic significance, as supported by prior literature [1, 6].

At the time of data extraction, 65 healthcare organizations across the U.S. had contributed eligible patient data. A comprehensive list of ICD‐10‐CM codes and temporal definitions used in the cohort construction is provided in Supporting Information S1: Table S1.

### 2.3 Outcomes

The primary outcome was the 1‐year risk of a composite of all‐cause death, stroke, acute myocardial infarction (AMI), and recurrent CA.

Secondary outcomes included the individual components of the primary outcome.

Each outcome was assessed with follow‐up beginning on day 3 after the index event to avoid capturing events related to the initial hospitalization and to minimize immortal time bias with the observation window extended through day 367 to reflect 1‐year follow‐up.

Events were identified using ICD‐10‐CM codes (see Supporting Information S1: Table S2 for full code list).

### 2.4 Statistical Analysis

Baseline characteristics of patients who developed AF after CA and those who did not were compared and balanced using logistic regression and propensity score matching (PSM) at a 1:1 ratio. Matching was performed using the greedy nearest neighbour method, employing a calliper of 0.1.

The following covariates were included in the PSM model: age, sex, ethnicity, hypertension, diabetes mellitus, dyslipidaemia, obesity, ischemic heart disease, cardiomyopathies, heart failure, cerebral infarction, chronic kidney disease, cancer, liver disease and cardiovascular medications (beta‐blockers, ACE inhibitors, angiotensin receptor blockers, calcium channel blockers, antianginals, lipid‐lowering agents, antiplatelets, antiarrhythmics, and diuretics). These covariates were selected based on their potential association with both the development of AF and the clinical course after CA. The balance of demographics and clinical variables between the groups was assessed using Absolute Standardized Mean Differences (ASD), with an ASD of less than 0.1 indicating adequate balance.

Univariable Cox proportional hazards models, before and after PSM, were used to calculate hazard ratios (HRs) and 95% confidence intervals (95% CIs) for the risk of adverse events in patients who developed AF after CA compared to those who did not. Absolute event rates, expressed as events per 1000 person‐years, were calculated from observed event counts and follow‐up time and are reported for all primary and secondary outcomes both before and after PSM.

To limit the potential impact of residual confounding related to post–CA illness severity, we repeated the post‐PSM Cox regression analyses by additionally adjusting for established proxies of critical illness severity available in the TriNetX platform, specifically shock (ICD‐10‐CM: R57.x) and acute kidney injury (ICD‐10‐CM: N17.x), in addition to the covariates described above.

Separately, to further explore the potential impact of anticoagulation on stroke outcomes, we conducted an additional sensitivity analysis comparing patients with post–CA AF and documented oral anticoagulant exposure to patients with CA without AF. PSM was applied to balance baseline characteristics, and outcomes were assessed using the same analytical approach as in the primary analysis.

Aalen‐Johansen curves were used to represent the daily cumulative incidence of secondary outcomes, accounting for all‐cause death as a competing risk, in patients who developed AF after CA compared to those who did not.

To validate the exposure definition, the 1‐year incidence of AF was assessed among patients who did not develop AF within 2 days after CA. This analysis aimed to exclude significant contamination by delayed‐onset AF. Incidence was estimated using Kaplan–Meier survival analysis and quantified by number of episodes within 1‐year from the start of the observation period. To further assess the robustness of the exposure definition, sensitivity analyses were performed using extended temporal windows of 3 and 7 days after CA to define post–CA AF. For each alternative window, cohorts were reconstructed, PSM was repeated using the same covariates as in the primary analysis, and outcomes were reassessed using identical statistical methods.

In the primary analysis, the proportional hazards assumption was tested for each outcome using Schoenfeld residuals and corresponding Chi‐square (*χ*²) tests. Further details on the performance and interpretation of this test can be found under the “Supporting Information S1: Methods” section of the Supporting Information S1: Material.

When the proportional hazards assumption was not met in the primary analysis, we performed additional analyses subdividing the 1‐year follow‐up period into two phases: an early phase (the first 30 days) and a late phase (from day 31 to the end of the year). We then reassessed the risk using Cox regression and re‐tested the proportional hazards assumption for each phase.

Pre‐specified subgroup analyses were performed to explore potential effect modification. Subgroups were defined according to: (1) patient characteristics, including age (patients aged ≥ 75 years vs. < 75 years) and sex (female vs. male); (2) cardiovascular comorbidities, including the presence or absence of hypertension (ICD‐10‐CM: I10), diabetes mellitus (ICD‐10‐CM: E08–E13), and heart failure (ICD‐10‐CM: I50.x); and (3) cardiac arrest features, based on the presence or absence of shockable rhythms such as ventricular tachycardia (ICD‐10‐CM: I47.2) or ventricular fibrillation (ICD‐10‐CM: I49.01). For each subgroup comparison, separate propensity score matching, and survival analyses were performed using the same matching criteria and statistical methods described for the primary analysis.

The construction of each cohort and the corresponding ICD‐10‐CM codes for inclusion and exclusion are reported in Supporting Information S1: Table S1.

All statistical analyses were performed with the TriNetX analytics platform, which integrates R (v3.2‐3) and Python 3.7 libraries (scikit‐learn for logistic models; survival for Cox analysis). TriNetX does not replace missing values. All *p*‐values were two‐tailed, with statistical significance set at *p* < 0.05.

## 3 Results

**Figure 1:**
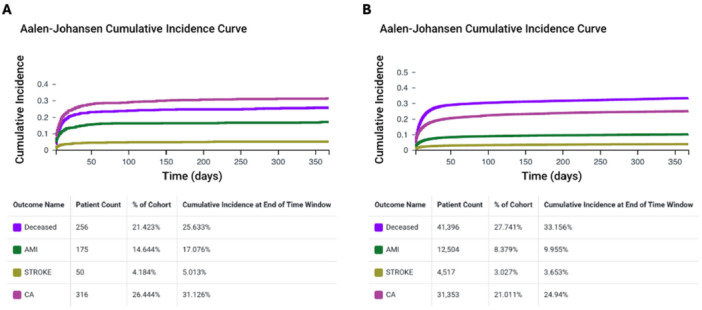
Aalen‐Johansen curves before propensity score matching for the cumulative incidence of primary and secondary outcomes in patients who developed AF after CA (Panel A) and who did not (Panel B). AF, atrial fibrillation; AMI, acute myocardial infarction; CA, cardiac arrest.

The final cohort included 1221 patients with post‐CA AF (mean age 65 ± 14.9 years; 33.8% female) and 149 222 patients without post‐CA AF (mean age 57.9 ± 17.6 years; 39.3% female); study flow‐chart is reported in Supporting Information S1: Figure 1.

Before PSM, patients with post‐CA AF were older, more frequently male, and exhibited a higher burden of cardiovascular comorbidities and related pharmacological treatments compared to those without AF (Table 1).

**Table 1:** Baseline characteristics of patients who developed AF after CA (Post‐CA AF) compared to those who did not (No post‐CA AF).

	Pre PSM			After PSM		
	Post CA‐AF (*n *= 1221)	No post CA‐AF (*n *= 149222)	ASD	Post CA‐AF (*n *= 1221)	No post CA‐AF (*n* = 1221)	ASD
Age at Index, mean ± SD	65.0 + /−14.9	57.9 + /−17.6	0.437	65.0 + /−14.9	64.7 + /−15.2	0.021
Female, *n* (%)	413 (33.8)	58601 (39.3)	0.113	413 (33.8)	438 (35.9)	0.043
Not Hispanic or Latino, *n* (%)	891 (73.0)	100 513 (67.4)	0.123	891 (73.0)	880 (72.1)	0.020
Ischemic heart disease, *n* (%)	621 (50.9)	333 499 (22.4)	0.617	621 (50.9)	671 (55.0)	0.082
Diabetes mellitus, *n* (%)	385 (31.5)	34 509 (23.1)	0.189	385 (31.5)	431 (35.3)	0.080
Hypertension, *n* (%)	591 (48.4)	57 981 (38.9)	0.193	591 (48.4)	668 (54.7)	0.126
Hyperlipidemia, *n* (%)	397 (32.5)	36 405 (24.4)	0.181	397 (32.5)	429 (35.1)	0.055
Heart failure, *n* (%)	436 (35.7)	24 456 (16.4)	0.451	436 (35.7)	436 (35.7)	< 0.001
Cerebral infarction, *n* (%)	108 (8.8)	7584 (5.1)	0.148	108 (8.8)	120 (9.8)	0.034
Cardiomyopathies, *n* (%)	115 (9.4)	9961 (6.7)	0.101	115 (9.4)	125 (10.2)	0.028
Obesity, *n* (%)	161 (13.2)	15 857 (10.6)	0.079	161 (13.2)	157 (12.9)	0.010
Chronic kidney disease, *n* (%)	296 (24.2)	24 900 (16.7)	0.188	296 (24.2)	334 (27.4)	0.071
Cancer, *n* (%)	236 (19.3)	28 151 (18.9)	0.012	236 (19.3)	268 (21.9)	0.065
Liver disease, *n* (%)	218 (17.9)	13 598 (9.1)	0.258	218 (17.9)	229 (18.8)	0.023
Beta blockers, *n* (%)	551 (41.9)	48 652 (32.6)	0.192	511 (41.9)	570 (46.7)	0.097
Antiarrhythmics, *n* (%)	681 (55.8)	51 185 (34.3)	0.442	681 (55.8)	752 (61.6)	0.118
Diuretics, *n* (%)	466 (38.2)	42 024 (28.2)	0.214	466 (38.2)	500 (41.0)	0.057
Calcium channel blockers, *n* (%)	330 (27.0)	31 874 (21.4)	0.133	330 (27.0)	375 (30.7)	0.081
ACE inhibitors, *n* (%)	240 (19.7)	27 102 (18.2)	0.038	240 (19.7)	288 (23.6)	0.096
Angiotensin II inhibitor, *n* (%)	153 (12.5)	16 825 (11.3)	0.039	153 (12.5)	184 (15.1)	0.074
Antianginals, *n* (%)	301 (24.7)	22 796 (15.3)	0.236	301 (24.7)	330 (27.0)	0.054
Lipid‐lowering agents, *n* (%)	482 (39.5)	42 619 (28.6)	0.232	482 (39.5)	519 (42.5)	0.062
Antiplatelets, *n* (%)	551 (45.1)	40 690 (27.3)	0.378	551 (45.1)	601 (49.2)	0.082

Abbreviations: ACE, angiotensin‐converting enzyme; AF indicates, atrial fibrillation; ASD, absolute standardized mean difference; CA, cardiac arrest.

The number of primary and secondary outcomes, along with corresponding HRs for the comparison before and after PSM, are reported in Table 2.

**Table 2:** Risks of primary and secondary outcomes in patients who developed AF after CA (Post‐CA AF) and compared to those who did not (no Post‐CA AF).

	Pre PSM					Post PSM				
	Post‐CA AF (events)	Post‐CA AF (rate/1000 PY)	No Post‐CA AF (events)	No Post‐CA AF (rate/1000 PY)	HR (95% CI)	Post‐CA AF (events)	Post‐CA AF (rate/1000 PY)	No Post‐CA AF (events)	No Post‐CA AF (rate/1000 PY)	HR (95% CI)
Composite	817	667.6	89 770	601.2	1.294 (1.208–1.387)	817	667.6	778	636.1	1.251 (1.134–1.381)
All‐cause death	461	375.5	55 938	374.6	1.003 (0.915–1.099)	461	375.5	511	374.6	0.935 (0.825–1.061)
Stroke	99	79.9	7295	48.4	1.713 (1.405–2.089)	99	79.9	82	55.2	1.305 (0.974–1.748)
Myocardial infarction	228	184.4	16 368	99.9	1.858 (1.631–2.118)	228	184.4	161	130.1	1.593 (1.302–1.950)
Recurrent CA	487	397.7	45 156	287.0	1.461 (1.336–1.597)	487	397.7	382	305.5	1.482 (1.296–1.695)

Abbreviations: AF, atrial fibrillation; CA, cardiac arrest; CI, confidence intervals; HR, hazard ratio; PSM, propensity score matching.

In the unmatched cohort, post‐CA AF patients showed a higher risk of the composite outcome (HR 1.417, 95% CI 1.298–1.547), stroke (HR 1.531, 95% CI 1.225–1.914), AMI (HR 1.742, 95% CI 1.516–2.001), and recurrent CA (HR 1.389, 95% CI 1.263–1.527) compared to those who did not develop AF (Table 2). No statistically significant difference was observed for all‐cause death (HR 1.024, 95% CI 0.933–1.124) (Table 2).

Aalen‐Johansen curves before PSM in patients with and without post‐CA AF are shown in Figure 1. The 1‐year cumulative incidences for primary and secondary outcomes were as follows: 26% and 33% for all‐cause death; 17% and 10% for myocardial infarction; 5% and 3% for stroke; 31% and 25% for recurrent CA, respectively (Figure 1A,B).

**Figure 2:**
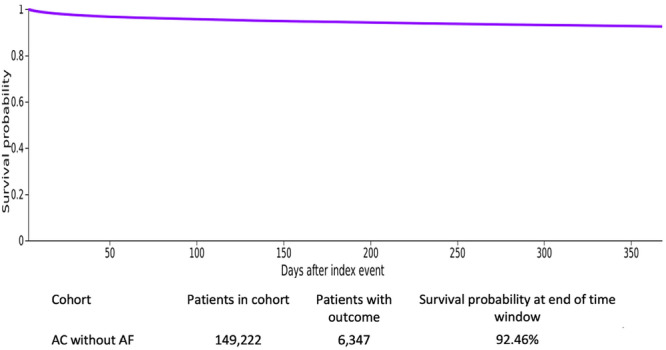
Kaplan–Meier curve representing the 1‐year probability of remaining free from AF among patients who did not develop AF within 2 days after CA. AF, atrial fibrillation; CA, cardiac arrest.

Among the 149 222 patients classified as No post‐CA AF, 6347 (7.54%) developed new‐onset AF between day 3‐ and 1‐year following CA. The estimated 1‐year probability of remaining free from AF was 92.46%, indicating minimal contamination of comparison group by delayed AF onset (Figure 2). This low incidence of delayed AF onset during follow‐up supports the specificity of the exposure definition and suggests minimal contamination of the comparison cohort by clinically unrecognized peri‐arrest AF.

Moreover, sensitivity analyses using extended exposure windows of 3 days and 7 days to define post–CA AF yielded results consistent with the primary 2‐day analysis (Supporting Information S1: Table S3).

After PSM, baseline characteristics—including demographics, cardiovascular comorbidities, and medication use—were well balanced across groups, with all ASD below 0.1 (Table 1).

In the matched cohort, post‐CA AF patients had a significantly higher risk of the composite outcome (HR 1.251, 95% CI 1.134–1.381), AMI (HR 1.593, 95% CI 1.302–1.950), and recurrent CA (HR 1.482, 95% CI 1.296–1.695) compared to those who did not develop AF (Table 2). No significant difference was observed for all‐cause death (HR 0.935, 95% CI 0.825–1.061), while a non‐significant trend toward increased risk was noted for stroke (HR 1.305, 95% CI 0.974–1.748) (Table 2).

When the post‐PSM analyses were repeated to further account for residual confounding related to illness severity by additionally adjusting for shock and acute kidney injury, the associations between post–CA AF and adverse outcomes remained directionally and quantitatively consistent (Supporting Information S1: Table S4).

Similarly, in sensitivity analyses evaluating the potential impact of anticoagulation, the association between post–CA AF and stroke remained directionally consistent with the primary analysis, with effect estimates of comparable magnitude, although statistical significance was attenuated (Supporting Information S1: Table S5).

**Figure 3:**
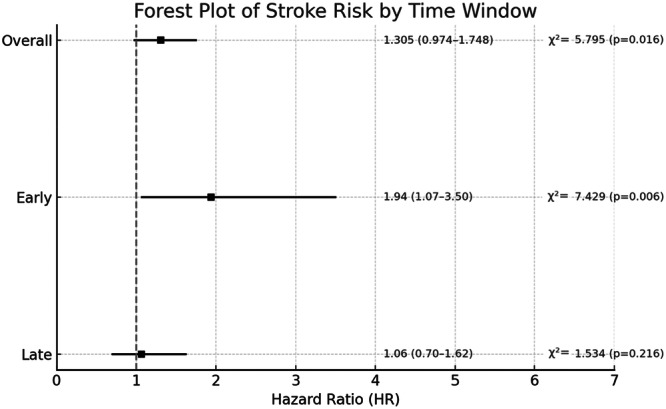
Risk of stroke in patients who developed AF after CA and who did not across overall, early (the first 30 days) and late (from day 31 to the end of the year) follow‐up phases. HR indicates Hazard Ratios‐ HR are reported with confidence intervals shown in parentheses. A high *χ*² value suggests a greater deviation from the expected values, indicating a potential violation of the proportional hazard assumption. Conversely, a small *χ*² value indicates that the observed residuals closely align with the expected values.

When testing the proportional hazards assumption for the 1‐year risk of all primary and secondary outcomes in patients with post‐CA AF compared to those without AF after PSM, no violations were observed for most outcomes. Specifically, the assumption held for the composite outcome (*χ*² = 3.522, *p*  =  0.061), all‐cause death (*χ*² = 0.584, *p*  =  0.445), AMI (*χ*² = 1.854, *p* = 0.173), and recurrent CA (*χ*² = 2.012, *p*  =  0.156) (Supporting Information S1: Figure 2). However, a statistically significant deviation from proportionality was observed for stroke (*χ*² = 5.795, *p*  =  0.016) (Figure 3).

When the follow‐up period was subdivided into an early phase (the first 30 days) and a late phase (from day 31 onwards), the risk of stroke was of greater magnitude during the early vs late phase of follow‐up (HR 1.94, 95% CI 1.07–3.50 vs. HR 1.06, 95% CI 0.70–1.62 for early vs. late period, respectively) (Figure 3).

The proportional hazard assumption for stroke was violated during the early phase (*χ*² = 7.429, *p* = 0.006) but satisfied during the late phase (*χ*² = 1.534, *p* = 0.216) (Figure 3).

### 3.1 Subgroup Analyses

**Figure 4:**
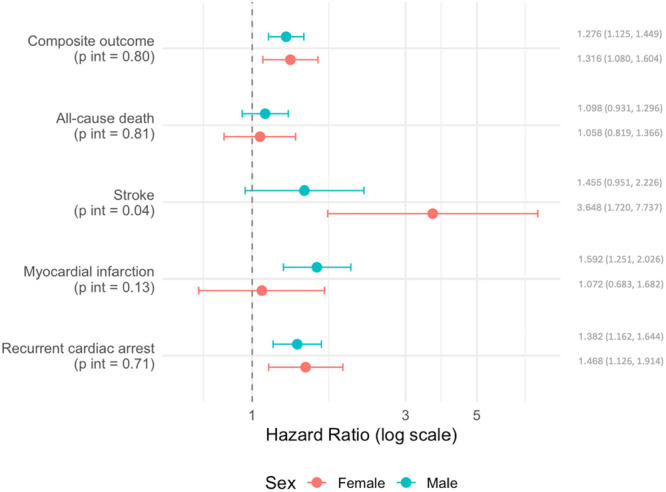
Risks of primary and secondary outcomes in patients who developed AF after CA compared to those who did not based on sex (females vs. males). HRs with 95% confidence intervals (CIs) shown in parentheses are shown for each outcome. *p* values for interaction between subgroups are reported in parentheses beneath each outcome label. AF, atrial fibrillation; CA, cardiac arrest.

No significant interactions were observed across age (patients aged ≥ 75 years vs. < 75 years) and presence or absence of hypertension, diabetes mellitus, heart failure, or shockable rhythms for any outcome (Supporting Information S1: Table S6). A significant interaction was detected for sex in relation to stroke (*p* for interaction = 0.04), with a more pronounced association between post‐CA AF and stroke observed among female patients (Supporting Information S1: Table S6; Figure 4).

## 4 Discussion

In our study, the principal findings are as follows: (1) Patients who developed AF after CA were older and had a higher burden of comorbidities and baseline cardiovascular risk; (2) Post‐CA AF was associated with an increased 1‐year risk of the composite outcome, driven primarily by higher risks of recurrent CA and AMI (3) The association between post‐CA AF and stroke was time‐ and sex‐dependent, with a higher risk observed during the early phase post‐CA and among female patients.

Patients who developed AF after CA were significantly older and more frequently male compared to those without AF. These demographic features are well‐established risk factors for incident AF in the general population. Age is the most thoroughly characterised [7, 8, 9], while male sex has been associated with a 1.5–2‐fold higher risk of developing AF, likely due to sex‐specific structural and electrophysiological differences [10]. In addition to these demographic factors, patients with AF also exhibited a higher burden of cardiovascular comorbidities, further supporting the close relationship between AF and cardiovascular risk factors [11, 12, 13].

In this context, although AF may represent a potential transient epiphenomenon of CA, it is likely to reflect an underlying, previously subclinical atrial disease—an arrhythmogenic substrate [14, 15, 16] unmasked or exacerbated by the acute physiological stress of CA.

Consistent with this hypothesis, patients who did not develop AF within the first 2 days after CA had a notably low prevalence of cardiovascular risk factors and exhibited a relatively low incidence of AF during the following year, suggesting the absence of an underlying arrhythmogenic substrate.

This interpretation is further supported by previous studies evaluating AF onset in acute settings such as cardiac surgery and sepsis, where AF was more frequent among older patients or those with pre‐existing hypertension, structural heart disease, or heart failure, all conditions closely associated with atrial remodelling [18, 19].

The occurrence of post‐CA AF, however, has important prognostic value, as demonstrated by the higher 1‐year risk of adverse events observed in this study—primarily driven by increased rates of recurrent CA, AMI, and stroke.

The high risk of recurrent CA observed in our study contrasts with findings from a retrospective study of emergency medical services patients, which reported no association between post‐ROSC AF and recurrent CA [19]. However, interpretation of that study should take into account its relatively small sample size (119 patients) and short follow‐up period (5–7 days), which may have limited its ability to detect meaningful associations. In contrast, our findings align with previous studies in broader AF populations, which consistently report an increased risk of sudden cardiac death. In community‐based cohorts such as ARIC and CHS, new‐onset AF was associated with nearly a twofold increase in the 1‐year risk of sudden cardiac death [20]. In a large nationwide French study, AF patients had an annual incidence of ventricular arrhythmias or sudden cardiac arrest of 2.23%, compared to 0.56% in non‐AF patients (HR 3.657; 95% CI, 3.604–3.711), over a median follow‐up of 5.4 years [21].

The high risk of ischaemic events associated with post‐CA AF is likely attributable to multiple factors. AF is a well‐recognized risk factor for arterial events, with studies like ARIC reporting a 1.4‐ to twofold increase in the risk of both myocardial infarction and stroke among patients with incident AF [22]. Evidence from community‐based cohorts and international registries has consistently shown that AF‐related myocardial ischaemia is particularly pronounced in patients with underlying structural heart disease or autonomic dysregulation [23, 24]. In the context of post‐CA, particularly within the first 72 h following return of spontaneous circulation, the myocardium exists in a highly vulnerable state—characterised by catecholamine excess, microvascular dysfunction, and oxygen supply–demand mismatch—which may further amplify the risk of ischaemic complications [2, 25].

In our cohort the risk of stroke was most pronounced within the first 30 days of follow up. These findings align with the pathophysiology of post‐CA syndrome, a condition defined by global ischemia–reperfusion injury, myocardial dysfunction, systemic inflammation, and autonomic imbalance—all of which peak in the first month after resuscitation and contribute to a transient period of heightened thrombotic vulnerability [2]. In this context, observational studies in OHCA (Out‐of‐Hospital Cardiac Arrest) survivors have shown a disproportionately high rate of early neurological and cardiovascular complications during this same period [25]. Incident AF further magnifies this early risk profile [26]. A large Danish nationwide cohort found that patients with new‐onset AF had a nearly fivefold increased risk of thromboembolic events within 30 days, particularly in the context of acute illness or hemodynamic compromise [27]. This risk is largely driven by atrial stunning, inflammation, and delays in anticoagulation initiation. Together, these mechanisms support a synergistic model, where the physiological stress of post‐CA syndrome, compounded by new‐onset AF, defines a critical window of ischemic instability [2], underscoring the need for intensified early‐phase monitoring, risk stratification, and timely management strategies during the first month post‐CA [29, 30, 31]. In this context, early post–CA AF may serve as a clinically relevant marker of heightened arrhythmic, ischemic, and thromboembolic vulnerability among survivors, supporting closer monitoring and individualized follow‐up strategies rather than routine escalation of specific therapeutic interventions.

The association between female sex and the risk of stroke in patients with post‐CA AF also warrants particular attention [31]. Although recent guidelines have suggested removing female sex from stroke risk stratification, that is, using the non‐sex CHA₂DS₂‐VASc score (ie. CHA₂DS₂‐VA) [33, 34], our findings suggest that caution may still be necessary as the impact of female sex on stroke risk in AF (and the predictive value of CHA₂DS₂‐VASc vs CHA₂DS₂‐VA), is dependent on the population studied [35, 36].

## 5 Limitations

Several limitations should be acknowledged. First, the retrospective observational design precludes causal inference and may be susceptible to residual confounding despite PSM. Second, exposure and outcome definitions relied on ICD‐10‐CM administrative codes, which may be affected by coding inaccuracies and do not allow assessment of key variables such as left ventricular function, AF duration, cardiac imaging, or hemodynamic status at AF onset. In addition, AF ascertainment relied on clinically documented diagnostic codes rather than continuous rhythm‐monitoring data, precluding assessment of AF burden, duration, or detection modality. As a result, brief, asymptomatic, or transient AF episodes that were not clinically recognized or coded may have been missed. This under‐ascertainment is expected to result in non‐differential misclassification, likely biasing associations toward the null. Importantly, our findings should therefore be interpreted as reflecting the prognostic impact of clinically recognized post–cardiac arrest AF, which is most relevant for real‐world risk stratification and follow‐up. Third, the 2‐day window to define post–CA AF, while consistent with prior literature, may have missed delayed‐onset cases or misclassified transient peri‐arrest arrhythmias, particularly in patients with prolonged intensive care unit stays or incomplete rhythm documentation. Fourth, mortality data were based on electronic health records and may underestimate deaths outside contributing institutions, leading to survival bias. Fifth, although propensity score matching achieved excellent balance across measured comorbidities and medications, unmeasured confounders—such as frailty, resuscitation quality, and specific post‐resuscitation interventions (e.g., sedation strategies, targeted temperature management, vasopressor type or dose)—could not be fully accounted for. While sensitivity analyses incorporating available proxies of illness severity yielded consistent results, residual confounding remains possible and should be considered when interpreting these findings. Sixth, AF subtypes (e.g., paroxysmal vs. persistent) could not be distinguished, though they may differ in prognosis and thromboembolic risk. Seventh, anticoagulation initiation, timing, persistence, and adherence could not be reliably characterized using the available data, particularly during the early post–CA phase. Although sensitivity analyses incorporating documented oral anticoagulant exposure yielded results consistent with the primary findings, residual confounding related to anticoagulation strategies cannot be excluded and should be considered when interpreting stroke‐related outcomes. Eighth, the TriNetX platform lacks detailed CA data, including arrest characteristics (e.g., initial rhythm, downtime, witnessed status), quality/duration of resuscitation, and post‐resuscitation care metrics—factors known to influence AF occurrence and outcomes. In addition, institution‐level characteristics (e.g., academic vs. community setting or geographic location) and variable‐level missingness could not be systematically assessed within the TriNetX platform, representing an inherent limitation of federated EHR‐based analyses. Ninth, subgroup analyses, while hypothesis‐generating, were underpowered to detect modest effect sizes and should be interpreted cautiously. Finally, although the TriNetX network is global, this analysis was conducted within the U.S. Collaborative Network, and the generalizability of our findings to other healthcare systems and populations warrants validation in independent CA cohorts.

## 6 Conclusion

Post–CA AF is associated with a higher risk of adverse events, including recurrent CA and myocardial infarction. The risk of stroke was more pronounced in women and during the early post‐CA period. Individualized risk stratification strategies that integrate temporal, demographic, and clinical factors to guide post‐CA follow‐up strategies are needed.

## Funding

The authors received no specific funding for this work.

## Conflicts of Interest

G.Y.H.L. has been a consultant and speaker for BMS/Pfizer, Boehringer Ingelheim, Anthos, and Daiichi‐Sankyo. No fees are directly received personally. All the disclosures happened outside the submitted work. He is a National Institute for Health and Care Research (NIHR) Senior Investigator Emeritus and co‐PI of the AFFIRMO project on multimorbidity in AF (grant agreement No 899871), TARGET project on digital twins for personalized management of atrial fibrillation and stroke (grant agreement No 101136244), and ARISTOTELES project on artificial intelligence for management of chronic long‐term conditions (grant agreement No 101080189), which are all funded by the EU's Horizon Europe Research and Innovation program. G.B. reported small speaker fees from Bayer, Boehringer Ingelheim, Boston, Daiichi Sankyo, Janssen, and Sanofi outside of the submitted work. G.B. is the Principal Investigator of the ARISTOTELES project (Applying aRtificial Intelligence to define clinical trajectorieS for personalized predicTiOn and early deTEction of comorbidity and muLti‐morbidity pattErnS) that received funding from the European Union within the Horizon 2020 research and innovation programme (grant no. 101080189). S.L.L. is supported by the National Medical Research Council Transitional Award; she has received research grants from Zoll Foundation, National University Health System, National Kidney Foundation of Singapore and Singapore Heart Foundation. She is an Associate Editor for Resuscitation and on the Editorial Board of Resuscitation Plus. The other authors did not report conflicts of interest to disclose outside of the submitted work.

## Supporting information


**Supporting Figure 1:** Flow chart of the study. CA indicates Cardiac Arrest; AF indicates Atrial Fibrillation. **Supporting Figure 2:** Risks of primary and secondary outcomes in patients with Post‐CA AF compared to those without. CA indicates Cardiac Arrest; AF indicates Atrial fibrillation; AMI indicates Acute Myocardial Infarction; CI indicates Confidence Intervals; HR, Hazard Ratio. A high χ2 suggests a greater deviation from the expected values, indicating a potential violation of the proportional hazard assumption. Conversely, a small χ2 value indicates that the observed residuals closely match the expected values.**Supporting Table S1:** ICD‐10‐CM codes for inclusion and exclusion criteria in patients with with Post‐CA AF compared to those without. In subgroup analyses stratified by age (≥75 and <75 years), the only criterion that differs from the main cohort definitions is the age threshold. CA indicates Cardiac Arrest; AF indicates Atrial fibrillation; ICD‐10 indicates International Classification of Diseases, 10th Revision (diagnosis codes); CPT indicates Current Procedural Terminology (procedure codes). **Supporting Table S2:** ICD‐10‐CM codes for the 1‐year risk of the composite outcome, all‐cause death, stroke, myocardial infarction and recurrent cardiac arrest. MI indicates myocardial infarction. CA indicates cardiac arrest. **Supporting Table S3:** Risks of primary and secondary outcomes after PSM in patients who developed AF after CA (Post‐CA AF) compared to those who did not (no Post‐CA AF) using alternative exposure windows of 3 days and 7 days after CA. **Supporting Table S4:** Risks of primary and secondary outcomes after PSM in patients who developed AF after CA (Post‐CA AF) compared to those who did not (no Post‐CA AF), with additional adjustment for acute kidney injury and shock. **Supporting Table S5:** Risks of primary and secondary outcomes in patients who developed AF after CA (Post‐CA AF) and have a documented oral anticoagulant exposure compared to those who did not (no Post‐CA AF). **Supporting Table S6:** Risks of primary and secondary outcomes in patients with Post‐CA AF compared to those without. Hazard Ratios (HR) are reported with 95% confidence intervals (CI) shown in parentheses. CA indicates Cardiac Arrest; AF indicates Atrial Fibrillation; ACD indicates All‐cause death; AMI indicates Acute Myocardial Infarction; HTA indicates Hypertension; HF indicates Heart Failure; P int indicates P for interaction.
