# Supplementary material for: Prognostic Impact of New‐Onset Atrial Fibrillation After Cardiac Arrest: Observations From a Federated Research Network
Source: J Cardiovasc Electrophysiol. 2026 Feb 10;37(4):758–67. doi: 10.1111/jce.70288 (PMC13069921; doi:10.1111/jce.70288)
Supplement: Supplementary file 1 — Supporting Figure 1: Flow chart of the study. CA indicates Cardiac Arrest; AF indicates Atrial Fibrillation. Supporting Figure 2: Risks of primary and secondary outcomes in patients with Post‐CA AF compared to those without. CA indicates Cardiac Arrest; AF indicates Atrial fibrillation; AMI indicates Acute Myocardial Infarction; CI indicates Confidence Intervals; HR, Hazard Ratio. A high χ2 suggests a greater deviation from the expected values, indicating a potential violation of the proportional hazard assumption. Conversely, a small χ2 value indicates that the observed residuals closely match the expected values.Supporting Table S1: ICD‐10‐CM codes for inclusion and exclusion criteria in patients with with Post‐CA AF compared to those without. In subgroup analyses stratified by age (≥75 and <75 years), the only criterion that differs from the main cohort definitions is the age threshold. CA indicates Cardiac Arrest; AF indicates Atrial fibrillation; ICD‐10 indicates International Classification of Diseases, 10th Revision (diagnosis codes); CPT indicates Current Procedural Terminology (procedure codes). Supporting Table S2: ICD‐10‐CM codes for the 1‐year risk of the composite outcome, all‐cause death, stroke, myocardial infarction and recurrent cardiac arrest. MI indicates myocardial infarction. CA indicates cardiac arrest. Supporting Table S3: Risks of primary and secondary outcomes after PSM in patients who developed AF after CA (Post‐CA AF) compared to those who did not (no Post‐CA AF) using alternative exposure windows of 3 days and 7 days after CA. Supporting Table S4: Risks of primary and secondary outcomes after PSM in patients who developed AF after CA (Post‐CA AF) compared to those who did not (no Post‐CA AF), with additional adjustment for acute kidney injury and shock. Supporting Table S5: Risks of primary and secondary outcomes in patients who developed AF after CA (Post‐CA AF) and have a documented oral anticoagulant exposure compared to those who did [file JCE-37-758-s001.docx]

**Prognostic impact of new-onset atrial fibrillation after cardiac arrest:**

**observations from a federated research network**

Enrico Tartaglia, M. Alobaida, Tommaso Bucci, Michele Rossi, Amir Askarinejad, Ho Man Lam, M. Kaskal, Andrea Galeazzo Rigutini, Shir Lynn Lim, Giuseppe Boriani, Gregory Y. H. Lip

Supplementary material

***Supplementary Methods***

TriNetX Database

The TriNetX data are collected from member healthcare organizations (HCO) and originates from their primary electronic health records (EHR) system. A typical HCO is a large academic health center with data coming from majority of its affiliates. A single HCO frequently has more than one facility, including main and satellite hospitals. The data are stored on the TriNetX database via a physical server at the institution’s data centre or a virtual hosted appliance. The TriNetX platform comprises of a series of these appliances connected into a federated network. This network can broadcast queries to each appliance. Results are subsequently collected and aggregated. Once the data are sent to the network, it is mapped to a standard and controlled set of clinical terminologies and undergoes a data quality assessment including ‘data cleaning’ that rejects records which do not meet the TriNetX quality standards. The TriNetX database performs internal and extensive data quality assessment with every refresh based on conformance, completeness, and plausibility (http://doi.org/10.13063/2327-9214.1244). HIPAA (Health Insurance Portability and Accountability Act) compliance of the clinical patient data is achieved using deidentification. Available data types within the network include demographics, diagnoses (represented by ICD-10-CM codes), procedures (coded in ICD-10-PCS or CPT), and measurements (coded to LOINC). While extensive information is provided about patients’ diagnoses and procedures, other variables (such as socioeconomic and lifetime factors are not comprehensively represented). The advantage of EHR data over insurance claim data is that both insured and uninsured patients are included. An advantage of EHR data over survey data is that the former represents the diagnostic rates in the population presenting to healthcare facilities. This provides an accurate account of the burden of specific diagnoses on healthcare systems. One primary limitation of relying on diagnoses is that they do not account for undiagnosed patients who might have a condition but have not yet received medical support. Another general limitation of EHR data is that a patient may be seen in different HCO for different components of their care. If one HCO is not part of the federated network, then part of their medical records may not be available. Using a network of healthcare organizations, rather than a single site, limits this possibility but does not fully remove it.

Propensity Score Matched Analyses were performed using logistic regression [Logistic Regression from the scikit-learn package in Python (version 3.7)]. TriNetX performed a 1:1 greedy nearest neighbor matching model with a caliper of 0.1 pooled standard deviations. To eliminate bias resulting from nearest neighbor algorithms, the rows were randomized. Any baseline characteristic with a standardized mean difference between cohorts lower than 0.1 was deemed well matched. (<https://www.tandfonline.com/doi/full/10.1080/00273171.2011.568786>).

Assessment of the Proportional Hazards Assumption

To evaluate whether the proportional hazards assumption was satisfied in the Cox regression models, we conducted a Chi-square (χ²) test based on Schoenfeld residuals. These tests examine whether the relationship between the associated variables and the hazard function remains stable over time. The null hypothesis posits that the effect of OAC discontinuation on the hazards of primary outcomes is constant throughout the study period. The χ² statistic measures the discrepancy between the observed and expected Schoenfeld residuals. A higher χ² value indicates a greater divergence from the expected values, suggesting a potential violation of the proportional hazards assumption. Conversely, a lower χ² value implies that the observed residuals closely align with the expected values, supporting the assumption. The p-value, derived from the χ² statistic, reflects the likelihood of observing these deviations under the null hypothesis. A p-value greater than 0.05 suggests that the deviations are likely due to random variation, indicating that the proportional hazards assumption holds. In contrast, a p-value less than 0.05 implies that the observed deviations are unlikely to be random, indicating a violation of the proportional hazards assumption.

**Supplementary Table S1**. ICD-10-CM codes for inclusion and exclusion criteria in patients with with Post-CA AF compared to those without. In subgroup analyses stratified by age (≥75 and <75 years), the only criterion that differs from the main cohort definitions is the age threshold. CA indicates Cardiac Arrest; AF indicates Atrial fibrillation; ICD-10 indicates International Classification of Diseases, 10th Revision (diagnosis codes); CPT indicates Current Procedural Terminology (procedure codes).

**Main Cohort Definitions and Characteristics**:

| Feature | Description |
| --- | --- |
| Network Queried | US Collaborative Network |
| Number of Healthcare Organizations | 65 |
| Patients Included | Post–CA AF: 1,221; No Post–CA AF: 149,222 (before matching); 1,221 (matched) |
| Index Diagnosis | Cardiac arrest (ICD-10: I46) |
| AF Definition | New diagnosis of AF (ICD-10: I48) within 2 days after CA |
| AF History Exclusion | No history of AF prior to CA |
| Exclusion Criteria | Deceased before or at index |
| Observation Window | 3 to 367 days post-index |
| Age Restriction | ≥18 years |
| Outcomes Assessed | All-cause mortality, stroke, AMI, recurrent CA, composite outcome |
| AF Timing Constraint | AF within 2 days from CA only (vs. absence of AF up to 2 days) |

## **Subgroup Definitions:**

| Subgroup | Definition | ICD-10 Codes (if applicable) |
| --- | --- | --- |
| Age | ≥75 years vs. <75 years | N/A |
| Hypertension | Presence vs. absence of diagnosis | I10–I15 |
| Diabetes Mellitus | Presence vs. absence of diagnosis | E08–E13 |
| Heart Failure | Presence vs. absence of diagnosis | I50 |
| Shockable Rhythm | Presence vs. absence at CA presentation | ICD-10 not applicable – defined clinically |

**Supplementary Table S2.** ICD-10-CM codes for the 1-year risk of the composite outcome, all-cause death, stroke, myocardial infarction and recurrent cardiac arrest. MI indicates myocardial infarction. CA indicates cardiac arrest.

| **Outcome** | **ICD-10-CM Description** |
| --- | --- |
| *Composite outcome* | Deceased (demographic status field, no ICD-10-CM code) OR Cerebral infarction (I63) OR Acute myocardial infarction (I21) OR Cardiac arrest (I46) |
| *All-cause death* | Deceased (demographic status field, no ICD-10-CM code) |
| *Stroke* | Cerebral infarction (I63) |
| *MI* | Acute myocardial infarction (I21) |
| *Recurrent CA* | Cardiac arrest (I46) |

**Supplementary Figure 1.** Flow chart of the study. CA indicates Cardiac Arrest; AF indicates Atrial Fibrillation.

**
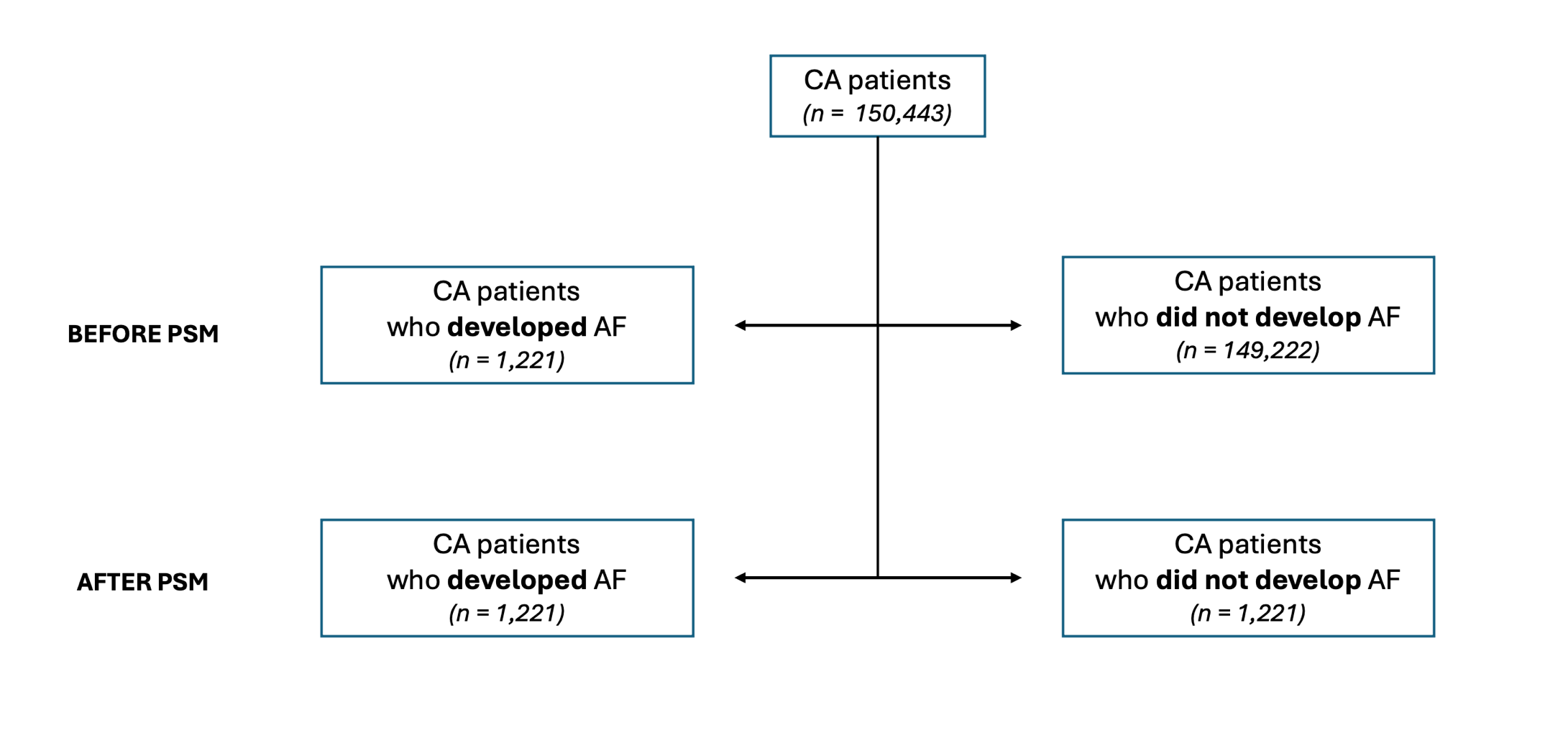
**

**Supplementary Figure 2.** Risks of primary and secondary outcomes in patients with Post-CA AF compared to those without. CA indicates Cardiac Arrest; AF indicates Atrial fibrillation; AMI indicates Acute Myocardial Infarction; CI indicates Confidence Intervals; HR, Hazard Ratio. A high χ2 suggests a greater deviation from the expected values, indicating a potential violation of the proportional hazard assumption. Conversely, a small χ2 value indicates that the observed residuals closely match the expected values.


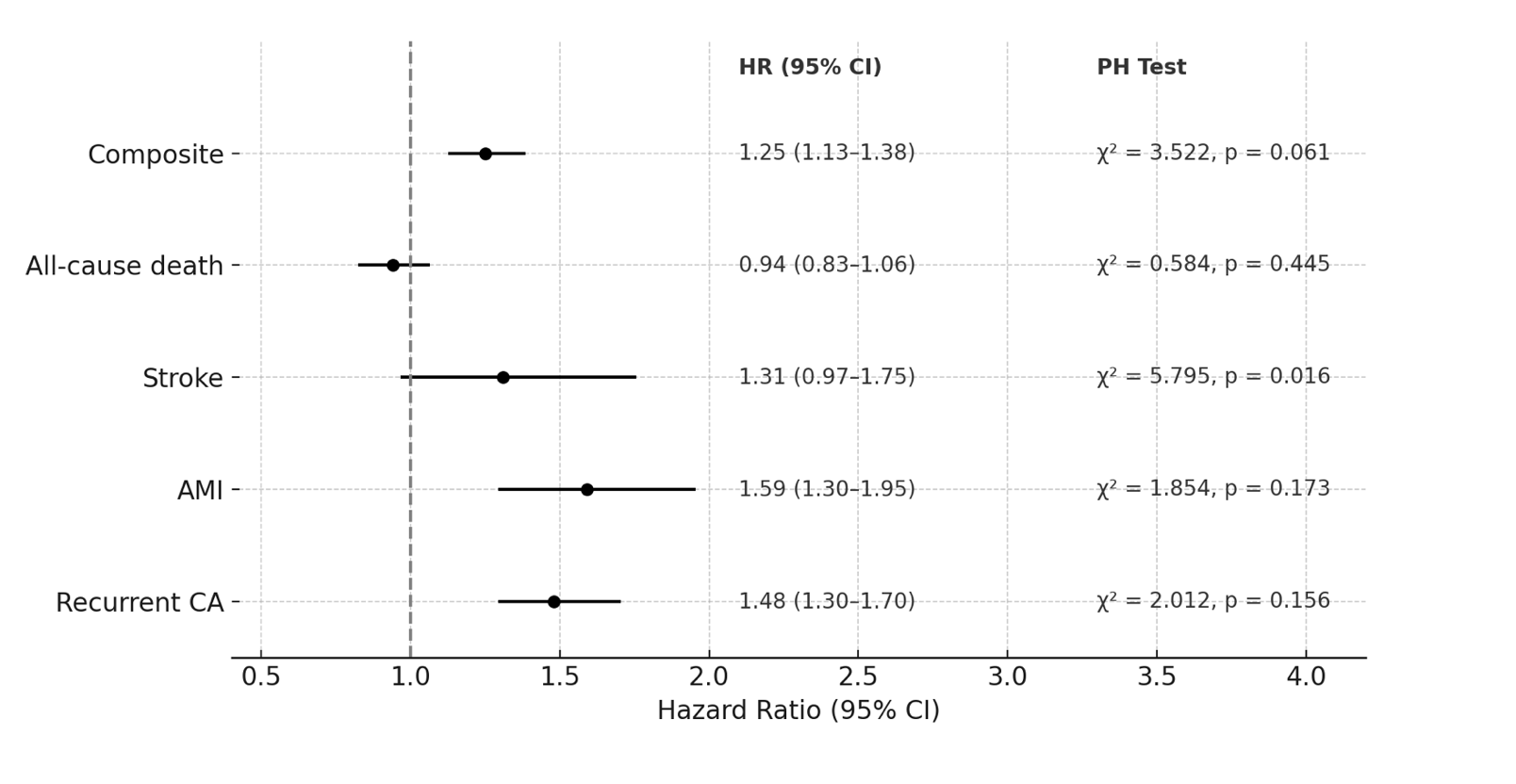


**Supplementary Table S3. Risks of primary and secondary outcomes after PSM in patients who developed AF after CA (Post-CA AF) compared to those who did not (no Post-CA AF) using alternative exposure windows of 3 days and 7 days after CA.**

AF indicates Atrial Fibrillation; CA indicates Cardiac Arrest. PSM indicates Propensity Score Matching. HR indicates Hazard Ratio. CI indicates Confidence Intervals

| **Outcome** | **3 days** | **7 days** |
| --- | --- | --- |
| **All-cause death** | HR 1.03 (95% CI 0.91–1.16) | HR 1.06 (95% CI 0.95–1.19) |
| **Stroke** | HR 1.48 (95% CI 1.12–1.96) | HR 1.31 (95% CI 1.06–1.64) |
| **Acute myocardial infarction** | HR 1.60 (95% CI 1.34–1.93) | HR 1.45 (95% CI 1.25–1.69) |
| **Recurrent cardiac arrest** | HR 1.39 (95% CI 1.23–1.57) | HR 1.44 (95% CI 1.30–1.60) |
| **Composite outcome** | HR 1.33 (95% CI 1.22–1.46) | HR 1.35 (95% CI 1.24–1.46) |

**Supplementary Table S4. Risks of primary and secondary outcomes after PSM in patients who developed AF after CA (Post-CA AF) compared to those who did not (no Post-CA AF), with additional adjustment for acute kidney injury and shock.**

AF indicates Atrial Fibrillation; CA indicates Cardiac Arrest. PSM indicates Propensity Score Matching. HR indicates Hazard Ratio. CI indicates Confidence Intervals

|  |  | **After PSM** | |  |
| --- | --- | --- | --- | --- |
|  | Post CA-AF  (1153) | No post CA-AF  (1153) | HR (95% CI) | |
| Composite | 777 | 738 | 1.266 (1.144, 1.400) | |
| All-cause death | 436 | 465 | 0.984 (0.864, 1.121) | |
| Stroke | 91 | 71 | 1.386 (1.016, 1.890) | |
| Myocardial Infarction | 223 | 186 | 1.328 (1.093, 1.614) | |
| Recurrent CA | 472 | 388 | 1.434 (1.253, 1.640) | |

**Supplementary Table S5. Risks of primary and secondary outcomes in patients who developed AF after CA (Post-CA AF) and have a documented oral anticoagulant exposure compared to those who did not (no Post-CA AF).**

AF indicates Atrial Fibrillation; CA indicates Cardiac Arrest. PSM indicates Propensity Score Matching. HR indicates Hazard Ratio. CI indicates Confidence Intervals

|  |  | **Pre PSM** | |  |  | **After PSM** | |  |
| --- | --- | --- | --- | --- | --- | --- | --- | --- |
|  | Post CA-AF  (919) | No post CA-AF  (151731) | HR (95% CI) | | Post CA-AF  (898) | No post CA-AF  (898) | HR (95% CI) | |
| Composite | 640 | 93058 | 1.339 (1.239, 1.447) | | 623 | 573 | 1.325 (1.182, 1.484) | |
| All-cause death | 351 | 57031 | 1.004 (0.904, 1.115) | | 344 | 368 | 0.955 (0.825, 1.107) | |
| Stroke | 74 | 7555 | 1.665 (1.324, 2.093) | | 71 | 55 | 1.383 (0.972, 1.966) | |
| Myocardial Infarction | 204 | 18070 | 2053 (1.788, 2.357) | | 201 | 112 | 2.072 (1.644, 2.612) | |
| Recurrent CA | 395 | 45184 | 1.475 (1.336, 1.629) | | 386 | 306 | 1.472 (1.267, 1.711) | |

**Supplementary Table S6.** Risks of primary and secondary outcomes in patients with Post-CA AF compared to those without. Hazard Ratios (HR) are reported with 95% confidence intervals (CI) shown in parentheses. CA indicates Cardiac Arrest; AF indicates Atrial Fibrillation; ACD indicates All-cause death; AMI indicates Acute Myocardial Infarction; HTA indicates Hypertension; HF indicates Heart Failure; P int indicates P for interaction

|  |  | **Composite** | |  | **ACD** | |  | **Stroke** | |  | **AMI** | |  | **Recurrent CA** | |
| --- | --- | --- | --- | --- | --- | --- | --- | --- | --- | --- | --- | --- | --- | --- | --- |
| **Subgroups** | HR (95% CI) | | *P int* | HR (95% CI) | | *P int* | HR (95% CI) | | *P int* | HR (95% CI) | | *P int* | HR (95% CI) | | *P int* |
| ≥ 75 years | 1.259 (1.081, 1.467) | | 0.76 | 0.992 (0.824, 1.194) | | 0.80 | 1.549 (0.916, 2.589) | | 0.43 | 1.541 (1.106, 2.146) | | 0.34 | 1.430 (1.157, 1.767) | | 0.98 |
| < 75 years | 1.299 (1.140, 1.481) | |  | 0.859 (0.803, 1.145) | |  | 2.016 (1.328, 3.060) | |  | 1.897 (1.448, 2.486) | |  | 1.435 (1.208, 1.705) | |  |
| Females | 1.316 (1.080, 1.604) | | 0.80 | 1.058 (0.819, 1.366) | | 0.81 | 3.648 (1.720, 7.737) | | 0.04 | 1.072 (0.683, 1.682) | | 0.13 | 1.468 (1.126, 1.914) | | 0.71 |
| Males | 1.276 (1.125, 1.449) | |  | 1.098 (0.931, 1.296) | |  | 1.455 (0.951, 2.226) | |  | 1.592 (1.251, 2.026) | |  | 1.382 (1.162, 1.644) | |  |
| HTA | 1.314 (1.294, 1.334) | | 0.46 | 0.967 (0.814, 1.149) | | 0.44 | 1.379 (0.957, 1.987) | | 0.58 | 1.441 (1.101, 1.885) | | 0.31 | 1.424 (1.308–1.589) | | 0.15 |
| No HTA | 1.449 (1.251, 1.678) | |  | 1.071 (0.882, 1.300) | |  | 1.664 (0.959, 2.887) | |  | 1.780 (1.308, 2.420) | |  | 1.722 (1.414, 2.095) | |  |
| Diabetes | 1.225 (1.035, 1.449) | | 0.18 | 0.941 (0.765, 1.157) | | 0.67 | 2.399 (1.375, 4.184) | | 0.51 | 1.398 (1.004, 1.947) | | 0.29 | 1.421 (1.123, 1.798) | | 0.19 |
| No Diabetes | 1.380 (1.220, 1.561) | |  | 1.006 (0.855, 1.185) | |  | 1.649 (1.094, 2.487) | |  | 1.771 (1.366, 2.296) | |  | 1.632 (1.381, 1.928) | |  |
| HF | 1.339 (1.145, 1.567) | | 0.71 | 0.933 (0.766, 1.137) | | 0.75 | 1.988 (1.255, 3.148) | | 0.71 | 1.633 (1.209, 2.204) | | 0.73 | 1.505 (1.218, 1.859) | | 0.71 |
| No HF | 1.288 (1.128, 1.469) | |  | 0.974 (0.821, 1.156) | |  | 1.748 (1.074, 2.846) | |  | 1.761 (1.303, 2.380) | |  | 1.586 (1.321, 1.905) | |  |
| Shockable | 1.277 (1.089, 1.499) | | 0.16 | 0.985 (0.777, 1.249) | | 1.00 | 1.255 (0.770, 2.044) | | 0.54 | 1.278 (0.989, 1.652) | | 0.94 | 1.323 (1.083, 1.616) | | 0.45 |
| No Shockable | 1.101 (0.963, 1.259) | |  | 0.985 (0.839, 1.157) | |  | 1.537 (0.996, 2.372) | |  | 1.301 (0.919, 1.842) | |  | 1.191 (0.987, 1.437) | |  |
